# Waist-to-height ratio and body roundness index: superior predictors of insulin resistance in Chinese adults and take gender and age into consideration

**DOI:** 10.3389/fnut.2024.1480707

**Published:** 2024-12-18

**Authors:** Anxiang Li, Yunwei Liu, Qi Liu, You Peng, Qingshun Liang, Yiming Tao, Yunyi Liu, Chongsong Cui, Qiqi Ren, Yingling Zhou, Jieer Long, Guanjie Fan, Qiyun Lu, Zhenjie Liu

**Affiliations:** ^1^Guangdong Provincial Hospital of Chinese Medicine, Guangzhou, China; ^2^The Second Affiliated Hospital of Guangzhou University of Chinese Medicine, Guangzhou, China; ^3^School of Second Clinical Medicine, Guangzhou University of Chinese Medicine, Guangzhou, China; ^4^Sun Yat-sen University, Guangzhou, China

**Keywords:** anthropometric indicators, insulin resistance, obesity, risk prediction, waist-to-height ratio, body roundness index

## Abstract

**Background and objectives:**

Metabolic disease has become a global health concern, and insulin resistance (IR) is a crucial underlying mechanism in various metabolic diseases. This study aims to compare the ability of seven anthropometric indicators in predicting IR in the Chinese population, and to find more sensitive and simple anthropometric indicator for early identification of IR.

**Methods:**

This prospective cross-sectional study obtained participants’ medical history, anthropometric indicators, and serum samples from three hospitals in China. Various anthropometric indicators were calculated, including body mass index (BMI), Waist-to-hip ratio (WHR), waist-to-height ratio (WtHR), conicity index (CI), A Body Shape Index (ABSI), body roundness index (BRI), abdominal volume index (AVI). The evaluation of IR is performed using the homeostasis model assessment-insulin resistance (HOMA-IR). Logistic regression analysis examined the relationship between indicators and HOMA-IR. The ability of the anthropometric indicators to predict IR was analyzed using the receiver operating characteristic (ROC) curve. Additionally, a stratified analysis was performed to evaluate the ability of the indicators in different age and gender groups.

**Results:**

The study included 1,592 adult subjects, with 531 in the non-IR group and 1,061 in the IR group. After adjusting for confounding factors, the anthropometric indicators showed a positive correlation with IR in the general population and across different genders and age groups (OR > 1, *p* < 0.05), except for ABSI. In the ROC curve analysis, WtHR and BRI had the highest AUC values of 0.711 for detecting IR. The optimal cut-off value for WtHR to diagnose IR was 0.53, while for BRI, it was 4.00. In the gender-stratified and age-stratified analysis, BMI, WtHR, BRI, and AVI all had AUC values >0.700 in females and individuals below 60.

**Conclusion:**

WtHR and BRI demonstrated a better ability to predict IR in the overall study population, making them preferred indicators for screening IR, and gender and age are important considerations. In the stratified analysis of different genders or age, BMI, WtHR, BRI, and AVI are also suitable for detecting IR in women or individuals under 60 years old in this study.

**Clinical trial registration:**

www.chictr.org.cn, ChiCTR2100054654.

## 1 Introduction

Metabolic diseases, including obesity, diabetes, hypertension, and dyslipidemia, have become global health concerns, with a notable upward trend in China due to lifestyle changes and an aging population (1–3). According to the latest data, the prevalence of obesity/overweight among Chinese adults is 50.7% (4), the prevalence of diabetes 11.2% (5), the prevalence of metabolic syndrome 33.9% (6). The prevalence of metabolic diseases will be an important factor in the incidence of cardiovascular diseases (7, 8). At the core of these metabolic disorders lies insulin resistance (IR), a condition characterized by decreased responsiveness of tissues such as the liver, skeletal muscle, and adipose tissue to the hormone insulin (9–11). Early detection and evaluation of IR have emerged as crucial strategies for preventing and managing these metabolic diseases as well as cardiovascular diseases.

Currently, the homeostasis model assessment-insulin resistance (HOMA-IR) is the most widely used method for evaluating IR in clinical practice (12). However, this method requires fasting blood glucose and insulin measurements, which can be challenging and resource-intensive, particularly in primary healthcare settings. Therefore, there is an urgent need to explore alternative, more accessible methods for identifying individuals at high risk of IR.

Anthropometric indicators have garnered significant attention as potential predictors of metabolic disorders. Body Mass Index (BMI), Waist-to-Hip Ratio (WHR), and Waist-to-Height Ratio (WtHR) are widely applied in clinical practice in China (13–15). More recently, researchers have developed novel indicators including the Conicity Index (CI) (16), A Body Shape Index (ABSI) (17), Body Roundness Index (BRI) (18), and Abdominal Volume Index (AVI) (19, 20), that have not yet been widely applied in clinical practice in China. These indicators, primarily calculated based on basic measurements of height, weight, waist circumference, and hip circumference, offer potential advantages in terms of accessibility and non-invasiveness, particularly in primary healthcare and epidemiological research settings (21–23). These anthropometric indicators will be beneficial for low-cost universal screening of IR and potential metabolic diseases (24, 25).

While recent studies have explored the utility of these anthropometric indicators in predicting various metabolic disorders such as diabetes (26), hypertension (27), cardiovascular disease (28–30), and metabolic syndrome (31), their relationship with IR in the Chinese population remains unclear. Moreover, the relative performance of these indicators in predicting IR across different age groups and genders has not been comprehensively evaluated.

Therefore, this study aims to compare the seven anthropometric indicators to find the most sensitive ones for early detection of IR in a Chinese population. Additionally, the study will establish optimal cut-off values for the most effective indicator(s) and assess differences in their predictive power across various age groups and genders. By addressing these objectives, it hopes to provide valuable insights for improving IR detection strategies, particularly in primary healthcare settings and epidemiological research in China. The findings could potentially inform the development of simple, cost-effective screening tools for identifying individuals at high risk of IR and associated metabolic disorders.

## 2 Methods

### 2.1 Study design and participants

This prospective cross-sectional study recruited participants from three hospitals in China: Guangdong Province Traditional Chinese Medical Hospital in southern China, and Jiangsu Provincial Hospital of Chinese Medicine in northern China, The Xinjiang Uygur Autonomous Region Institute of Traditional Chinese Medicine in northwest China between August 1, 2020, and December 31, 2021. The research recruited participants through poster advertisements in the outpatient and inpatient departments of three hospitals, and participants were enrolled a non-randomized continuous sampling method. In a quiet and private consultation room, the researcher collected questionnaire data from the participants through face-to-face interviews. The study was conducted following ethical standards and was approved by the Guangdong Province Traditional Chinese Medical Hospital (Approval No. BE2021-156-01). All participants provided written informed consent. The study was registered with the China Clinical Trial Registration Center (Registration Number: ChiCTR2100054654).

Inclusion criteria were: (1) age 18–90 years; (2) ability to provide informed consent. Exclusion criteria included: (1) hyperthyroidism or hypothyroidism; (2) secondary hyperlipidemia, hyperglycemia, or hypertension; (3) type 1 diabetes; (4) use of medications affecting weight (e.g., glucocorticoids, contraceptives, diet pills); (5) pregnancy or lactation; (6) acute infection; (7) severe heart, liver, or kidney insufficiency; (8) malignant tumors.

### 2.2 Anthropometric measurements and calculations

Trained researchers used standardized testing devices to collect anthropometric data. Height and weight were measured using the RGZ-120-RT integrated height and weight measuring tool (Xiheng brand). Waist and hip circumferences were measured using a non-shrinking elastic measuring tape. Body fat percentage (BFP) and visceral fat index (VFI) were measured using the HUAWEI TruFitTM (3 Pro).

Seven anthropometric indices were calculated using the following formulas:

(1) Body mass index (32) = weight (kg)/height^2^ (m);
(2) Waist-to-hip ratio (33) = waist circumference (cm)/hip circumference (cm);
(3) Waist-to-height ratio (34)=waist circumference (cm)/height (cm);
(4) Conicity index (16) =0.109^−1^ waist circumference (m)*(weight (kg)/height (m))^-1/2^;
(5) A body shape index (17) =waist circumference (m)/(BMI^2/3^ (kg/m^2^) *height ^1/2^ (m));
(6) Body Roundness Index (18) =364.2–365.5 (1-*π*^−2^ Waist ^2^ (m)*Height^−2^ (m))^1/2^;
(7) Abdominal volume index (35) = (2 (waist circumference)^2^ (cm) + 0.7 (waist circumference (cm)-hip circumference (cm))^2^)/1000.

### 2.3 Biochemical examination

Blood samples were collected after an 8-h fast and analyzed using the Cobas 8,000 biochemical analyzer for fasting blood glucose (FBG), lipid profile, and uric acid. Glycated hemoglobin was measured using the Sebia CAP instrument, and insulin levels were measured using the ATELLICO instrument.

### 2.4 Definition of IR and metabolic diseases

IR was evaluated using the HOMA-IR, calculated as: HOMA-IR = (FPG mmol/L) * (FINS mIU/L)/22.5. IR was defined as HOMA-IR ≥ 1.7, non-IR was defined as HOMA-IR < 1.7 (36, 37).

*Metabolic syndrome* (38): Three or more of the following criteria needed to be met: (1) abdominal obesity: waist circumference ≥ 90 cm for men and ≥ 85 cm for women; (2) hyperglycemia: fasting blood glucose ≥6.1 mmoL/L or 2 h postprandial blood glucose ≥7.8 mmoL/L and/or a diagnosis of diabetes with treatment; (3) hypertension: blood pressure ≥ 130/85 mmHg and/or confirmed hypertension on treatment; (4) fasting TG ≥ 1.70 mmoL/L; (5) fasting HDL-C < 1.04 mmoL/L. *Hypertension* (39): At least twice in the morning, blood pressure ≥ 140/90 mmHg and/or confirmed hypertension on treatment. *Obesity* (40): BMI ≥ 24.0 kg/m^2^ or waist circumference ≥ 90 cm for men and ≥ 85 cm for women. *Hyperlipidemia* (41): one the following criteria needed to be met: (1) fasting TG ≥ 2.3 mmoL/L; (2) fasting TC ≥ 6.2 mmoL/L; (3) fasting LDL-C ≥ 4.1 mmoL/L; (4) fasting HDL-C < 1.00 mmoL/L; (5) and/or confirmed hyperlipidemia on treatment. *Abnormal blood glucose* (38): fasting blood glucose ≥6.1 mmoL/L or two hour postprandial blood glucose ≥7.8 mmoL/L and/or a diagnosis of diabetes with treatment.

### 2.5 Statistical analysis

Sample Size Calculation: Assuming a type I error of 0.05, a power of 0.90, a AUC^0^ of 0.50, and an anticipated area under the ROC curve (AUC) of 0.60 to 0.7, we calculated a required sample size of 314 to1410 participants, and chosen the maximum sample size by using the pROC package in R. Assuming a response rate and efficient rate of 94%, therefore, the minimum sample calculation for this study is recalculated as 1,500.

All statistical analyses were performed using SPSS 22.0 (SPSS, Inc., Chicago, IL, USA). The normality of continuous variables was assessed using the Kolmogorov–Smirnov test, and homogeneity of variance was tested using Levene’s test. Normally distributed continuous variables were presented as mean ± standard deviation (SD) and compared using Student’s *t*-test. Non-normally distributed variables were presented as median (interquartile range) and compared using the Wilcoxon rank-sum test. Categorical variables were presented as numbers (percentages) and compared using the chi-square test.

Univariate and multivariate binary logistic regression analyses were performed to examine the association between anthropometric indicators and HOMA-IR. In the multivariate analysis, it adjusted the confounding variables including age, sex, smoking status, and alcohol consumption. Results were presented as odds ratios (ORs) with 95% confidence intervals (CIs).

The predictive ability of each anthropometric indicator for IR was evaluated using receiver operating characteristic (ROC) curve analysis. The area under the ROC curve (AUC) was calculated to assess the overall discriminative power of each indicator (42). An AUC > 0.700 was considered acceptable (43). The optimal cut-off value for each indicator was determined using the Youden index (sensitivity + specificity – 1) (43). Sensitivity, specificity, and Youden index were calculated for each cut-off value.

To examine potential differences in the predictive power of anthropometric indicators across different subgroups, we performed stratified analyses by gender (male vs. female) and age (<60 years vs. ≥60 years). For each subgroup, we conducted separate logistic regression and ROC curve analyses following the procedures described above. All statistical tests were two-tailed, and a *p*-value <0.05 was considered statistically significant.

## 3 Results

### 3.1 Clinical characteristics

**Figure 1:**
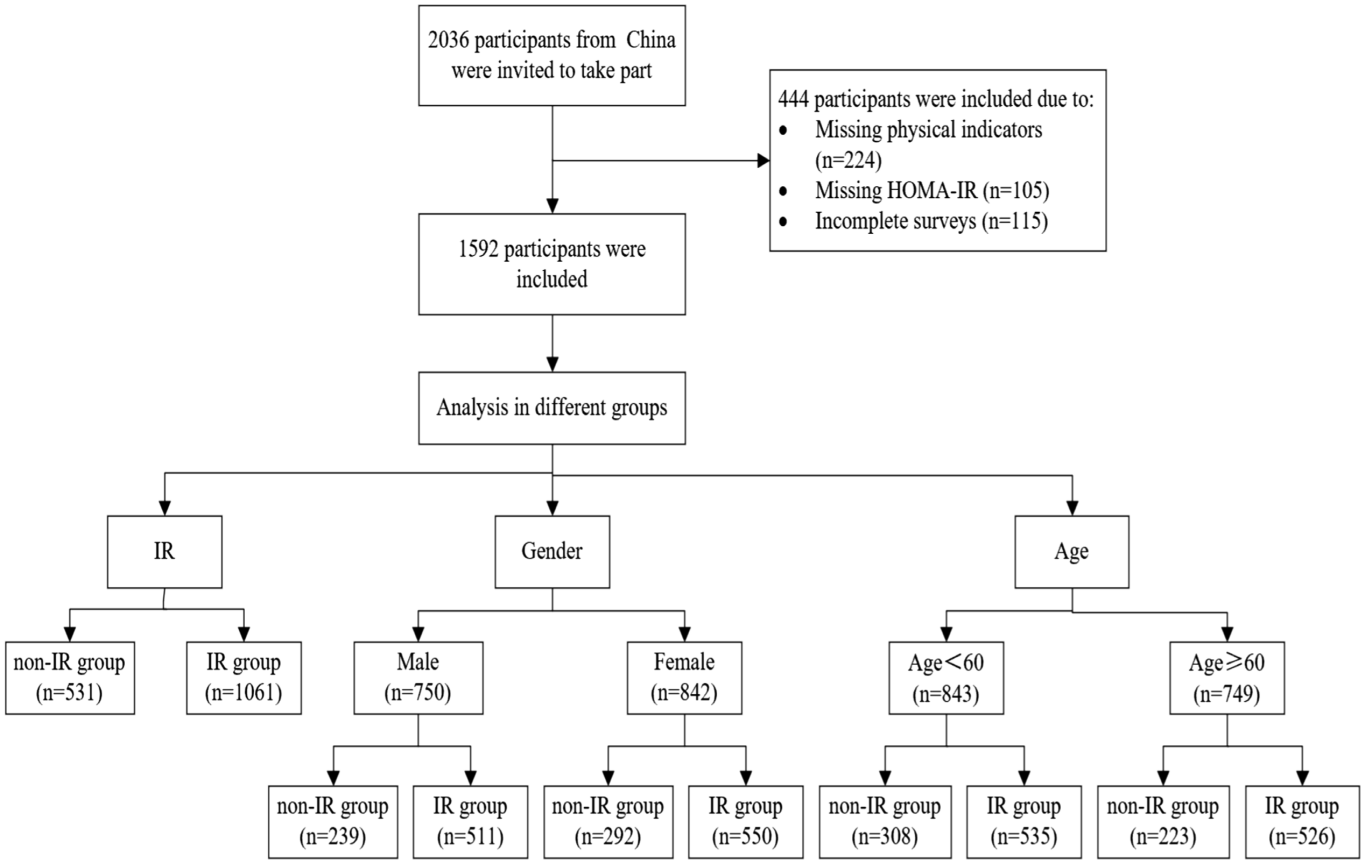
Participant flow chart.

A total of 2036 participants were involved in this study. Among them, 444 participants were excluded due to missing anthropometric measurements or HOMA-IR data, or incomplete surveys. Ultimately, 1952 participants (842 females and 750 males) were successfully included for the study and data analysis, of which 531 were categorized in the non-IR group and 1,061 in the IR group (shown in Figure 1). The weight, SBP, DBP, TC, TG, LDL-C, FBG, HbA1c, HOMA-IR, uric acid, BFP, and VFI in the IR group were all higher than those in the non-IR group (*p* < 0.001). Additionally, the values of BMI, WHR, WtHR, CI, ABSI, BRI, and AVI in the IR group were higher than those in the non-IR group (*p* < 0.001). The proportions of metabolic syndrome, Hypertension, Obesity, Hyperlipidemia, and Abnormal blood glucose were higher in the IR group compared to the non-IR group (*p* < 0.001). The primary characteristics of the overall study population, non-IR group, and IR group are detailed in Tables 1, 2.

**Table 1:** Basic characteristics according to HOMA-IR.

Variables	All (*n* = 1,592)	Non-IR^1^ group (*n* = 531)	IR group (*n* = 1,061)	*P-*value
Age (y^2^)	56.63 ± 14.62	54.68 ± 15.84	57.60 ± 13.88	<0.001
Gender: F^3^ (%)	842 (52.9%)	292 (55%)	550 (51.8%)	0.235
Height (cm)	164.11 ± 8.69	164.66 ± 8.66	163.84 ± 8.70	0.073
Weight (kg)	69.10 ± 13.44	65.05 ± 11.55	71.11 ± 13.86	<0.001
BMI^4^ (kg/m^2^)	25.54 ± 3.80	23.83 ± 3.07	26.36 ± 3.87	<0.001
SBP^4^ (mmHg)	130.23 ± 17.17	125.11 ± 16.77	132.78 ± 16.80	<0.001
DBP^5^ (mmHg)	78.13 ± 10.79	75.95 ± 10.68	79.21 ± 10.68	<0.001
TC^6^ (mmol/L)	4.64 ± 1.22	4.41 ± 1.11	4.76 ± 1.26	<0.001
TG^7^ (mmol/L)	1.92 ± 1.69	1.45 ± 1.19	2.16 ± 1.85	<0.001
LDL-C^8^ (mmol/L)	2.90 ± 0.99	2.68 ± 0.93	3.00 ± 1.00	<0.001
HDL-C^9^ (mmol/L)	1.21 ± 0.37	1.31 ± 0.38	1.16 ± 0.36	<0.001
FBG^10^ (mmol/L)	7.00 ± 2.62	5.66 ± 1.61	7.66 ± 2.76	<0.001
HbA1c (%)	7.53 ± 2.34	6.52 ± 1.69	8.04 ± 2.46	<0.001
Fasting insulin (pmol/L)	73.22 ± 60.51	31.99 ± 12.37	93.85 ± 64.36	<0.001
HOMA-IR^12^	3.40 ± 3.25	1.10 ± 0.38	4.55 ± 3.43	<0.001
Uric acid (mmol/L)	355.19 ± 107.75	327.36 ± 97.23	369.12 ± 110.08	<0.001
BFP^13^ (%)	29.55 ± 6.40	27.98 ± 6.54	30.34 ± 6.17	<0.001
VFI^14^	10.53 ± 4.90	8.83 ± 4.90	11.39 ± 4.68	<0.001
Drinker (%)	378 (23.7%)	111 (21%)	226 (21.3%)	0.553
Smoker (%)	337 (21.3%)	110 (20.7)	268 (25.3%)	0.045

^1IR, insulin resistance; 2y, years old; 3F, female; 4BMI, body mass index; 5SBP, systolic blood pressure; 6DBP, diastolic blood pressure; 7TC, total cholesterol; 8TG, triglyceride; 9LDL-C, low-density lipoprotein cholesterol; 10HDL-C, high-density lipoprotein cholesterol; 11FBG, fasting blood glucose; 12HOMA-IR, homeostasis model assessment-insulin resistance; 13BFP, body fat percentage; 14VFI, visceral fat index.^

**Table 2:** Anthropometric indicators and medical history of two groups.

Variables	All (*n* = 1,592)	non-IR group (*n* = 531)	IR group (*n* = 1,061)	*P-*value
BMI^1^	25.53 ± 3.80	23.83 ± 3.06	26.36 ± 3.86	<0.001
WHR^2^	0.945 ± 0.08	0.920 ± 0.09	0.957 ± 0.07	<0.001
WtHR^3^	0.557 ± 0.06	0.526 ± 0.06	0.572 ± 0.05	<0.001
CI^4^	1.297 ± 0.09	1.267 ± 0.10	1.311 ± 0.08	<0.001
ABSI^5^	0.082 ± 0.005	0.0815 ± 0.006	0.0830 ± 0.005	<0.001
BRI^6^	4.57 ± 1.34	3.93 ± 1.22	4.89 ± 1.28	<0.001
AVI^7^	16.98 ± 3.88	15.34 ± 3.65	17.81 ± 3.73	<0.001
Metabolic syndrome (%)	940 (59%)	196 (36.9%)	744 (70.1%)	<0.001
Hypertension (%)	785 (49.3%)	194 (36.6%)	591 (55.7%)	<0.001
Obesity (%)	1,076 (67.7%)	259 (49%)	817 (77.1%)	<0.001
Hyperlipidemia (%)	715 (45%)	168 (31.8%)	547 (51.6%)	<0.001
Abnormal blood glucose (%)	1,137 (71.4%)	263 (49.5%)	874 (82.4%)	<0.001

^1BMI, body mass index; 2WHR, waist-to-hip ratio; 3WtHR, waist-to-height ratio; 4CI, conicity index; 5ABSI, A Body Shape Index; 6BRI, body roundness index; 7AVI, abdominal volume index.^

### 3.2 Comparison of anthropometric indicators between 2 groups in different gender and age participants

In the analysis of different gender and age participants, the anthropometric indicators also showed differences in the non-IR and IR groups, displayed in Tables 3, 4 for details. Specifically, when comparing male and female subjects, the mean values of BMI, WHR, WtHR, CI, ABSI, BRI, and AVI were significantly higher in the IR group than in the non-IR group (*P* < 0.05). Researchers also examined the differences in these indicators based on age groups (subjects aged over 60 or less). Similar to the findings in gender comparison, except for ABSI, the mean values of BMI, WHR, WtHR, CI, BRI, and AVI in the IR group were higher than those in the non-IR group (*p* < 0.05), regardless of age.

**Table 3:** Comparison of anthropometric indicators between 2 groups in different gender.

Variables	Male (*n* = 750)			Female (*n* = 842)		
	Non-IR group (*n* = 239)	IR group (*n* = 511)	*P-*value	Non-IR group (*n* = 292)	IR group (*n* = 550)	*P-*value
BMI^1^	24.65 ± 2.95	26.62 ± 3.79	<0.001	23.23 ± 3.01	26.12 ± 3.91	<0.001
WHR^2^	0.945 ± 0.08	0.974 ± 0.06	<0.001	0.898 ± 0.10	0.941 ± 0.07	<0.001
WtHR^3^	0.513 ± 0.05	0.565 ± 0.05	<0.001	0.522 ± 0.06	0.579 ± 0.06	<0.001
CI^4^	1.28 ± 0.08	1.31 ± 0.07	<0.001	1.25 ± 0.11	1.30 ± 0.09	<0.001
ABSI^5^	0.082 ± 0.005	0.083 ± 0.004	0.024	0.080 ± 0.001	0.083 ± 0.001	<0.001
BRI^6^	4.01 ± 1.08	4.72 ± 1.16	<0.001	3.86 ± 1.32	5.04 ± 1.36	<0.001
AVI^7^	16.72 ± 3.39	18.75 ± 3.75	<0.001	14.20 ± 3.46	16.92 ± 3.49	<0.001

^1BMI, body mass index; 2WHR, waist-to-hip ratio; 3WtHR, waist-to-height ratio; 4CI, conicity index; 5ABSI, A Body Shape Index; 6BRI, body roundness index; 7AVI, abdominal volume index.^

**Table 4:** Comparison of anthropometric indicators between 2 groups in different age.

Variables	Age < 60 (*n* = 843)			Age ≥ 60 (*n* = 749)		
	Non-IR group (*n* = 308)	IR group (*n* = 535)	*P-value*	Non-IR group (*n* = 223)	IR group (*n* = 526)	*P-*value
BMI^1^	23.81 ± 3.14	27.04 ± 4.23	<0.001	23.93 ± 2.94	25.66 ± 3.31	<0.001
WHR^2^	0.901 ± 0.08	0.953 ± 0.07	<0.001	0.945 ± 0.10	0.960 ± 0.07	0.027
WtHR^3^	0.510 ± 0.05	0.565 ± 0.06	<0.001	0.548 ± 0.05	0.579 ± 0.05	<0.001
CI^4^	1.23 ± 0.09	1.28 ± 0.08	<0.001	1.31 ± 0.10	1.33 ± 0.07	0.002
ABSI^5^	0.079 ± 0.005	0.081 ± 0.005	<0.001	0.084 ± 0.007	0.085 ± 0.005	0.423
BRI^6^	3.62 ± 1.15	4.74 ± 1.31	<0.001	4.37 ± 1.18	5.03 ± 1.23	<0.001
AVI^7^	14.72 ± 3.62	18.00 ± 4.18	<0.001	16.13 ± 3.50	17.61 ± 3.22	<0.001

^1BMI, body mass index; 2WHR, waist-to-hip ratio; 3WtHR, waist-to-height ratio; 4CI, conicity index; 5ABSI, A Body Shape Index; 6BRI, body roundness index; 7AVI, abdominal volume index.^

### 3.3 Logistic regression analysis of anthropometric indicators and IR

Logistic regression analysis was performed to assess the relationship between the anthropometric indicators and IR. It was found that in the general population, both individually and after controlling for confounding factors such as age, gender, smoking, and drinking, IR was positively correlated with all anthropometric indicators (OR > 1, *p* < 0.05). When the analysis was performed separately for male and female subjects, after adjusting for confounding factors (age, smoking, drinking), IR was still found to be correlated with all the indicators except for ABSI (*p* < 0.05). It indicates that in both genders, these anthropometric indicators were associated with an increased risk of IR. Furthermore, the study also revealed that regardless of age and confounding factors (gender, smoking, drinking), IR was significantly correlated with BMI, WHR, WtHR, CI, BRI, and AVI (OR > 1, *p* < 0.05) in subjects of different age groups. These anthropometric indicators can serve as predictors for the presence of IR, independent of age and other potential confounders. Table 5 provides detailed information on these correlations.

**Table 5:** Logistic regression for anthropometric indicators and IR.

Index	All		Gender				Age			
	OR^◇^ (95% Cl)	OR^▲^ (95% Cl)	Male		Female		Age < 60		Age ≥ 60	
			OR^◇^ (95% Cl)	OR^◆^ (95% Cl)	OR^◇^ (95% Cl)	OR^◆^ (95% Cl)	OR^◇^ (95% Cl)	OR■ (95% Cl)	OR^◇^ (95% Cl)	OR■ (95% Cl)
BMI	1.24 (1.20, 1.29)	1.24 (1.20, 1.29)	1.20 (1.13, 1.27)	1.20 (1.14, 1.27)	1.28 (1.20, 1.33)	1.26 (1.22, 1.35)	1.29 (1.23, 1.36)	1.28 (1.22, 1.35)	1.20 (1.13, 1.27)	1.20 (1.13, 1.27)
WHR	327 (81, 1,320)	272 (61, 1,210)	335 (32, 3,421)	354 (33, 3,752)	437 (68, 2,794)	109 (15, 779)	2,491 (374, 1.6×10^4^)	2094 (270, 1.6×10^4^)	10.87 (1.31, 89.84)	13.47 (1.52, 118)
WtHR	1.2×10^6^ (1.4×10^5^, 1.0 ×10^7^)	1.6×10^6^ (1.7×10^5^, 1.6 ×10^7^)	4.2×10^5^ (1.4×10^4^, 1.2 ×10^7^)	6.8×10^5^ (2.0×10^4^, 2.2 ×10^7^)	2.5×10^6^ (1.6×10^5^, 3.8 ×10^7^)	1.3×10^6^ (6.4×10^4^, 2.7×10^7^)	1.0×10^7^ (5.6×10^5^, 2.0 ×10^8^)	8.2×10^6^ (4.1×10^5^, 1.6×10^8^)	6.3 ×10^4^ (2,344, 1.7 ×10^6^)	6.0×10^4^ (2,104, 1.7 ×10^6^)
CI	228 (65, 792)	214 (53, 852)	142 (18, 1,118)	315 (35, 2,809)	290 (60, 1,406)	84 (13, 525)	1,565 (253, 9,687)	1,055 (162, 6,854)	18.66 (2.82, 123)	20.18 (2.99, 136)
ABSI	2.986E+19 (2.539E+11,3.511E+27)	1.558E+15 (1.6×10^6^,1.4851E+24)	7.279E+15 (286, 1.851E+29)	3.067E+21 (9.5×10^6^, 9.808E+35)	1.374E+21 (1.002E+11,1.883E+31)	*5.0×10^7^ (0, 2.089E+19*)	6.511E+28 (3.798E+16, 1.116E+41)	3.136E+25 (1.433E+13, 6.862E+37)	*2.7×10^5^ (0.01, 1.685E+17)*	*6.7×10^5^ (0.01, 5.478E+17)*
BRI	1.96 (1.77, 2.18)	1.98 (1.77, 2.22)	1.86 (1.57, 2.20)	1.90 (1.59, 2.25)	2.04 (1.78, 2.33)	1.96 (1.69, 2.28)	2.24 (1.93, 2.60)	2.20 (1.90, 2.56)	1.66 (1.42, 1.95)	1.66 (1.41, 1.95)
AVI	1.22 (1.18, 1.27)	1.24 (1.19, 1.29)	1.19 (1.13, 1.26)	1.19 (1.13, 1.26)	1.29 (1.22, 1.36)	1.26 (1.20, 1.34)	1.27 (1.21, 1.33)	1.28 (1.21, 1.34)	1.16 (1.09, 1.22)	1.18 (1.11, 1.25)

◇ unadjusted; ▲ adjusted for age, gender, smoking, and drinking; ◆ adjusted for age, smoking, and drinking; ■ adjusted for gender, smoking, and drinking. Values of OR are not statistically significant when they are in italics.

### 3.4 Analysis of the AUC value of all anthropometric indicators in predicting IR

Based on the results, the study found that the AUC values, which indicate the accuracy of the anthropometric indicators in predicting IR, varied from 0.538 to 0.748 overall (Table 6). The analysis also revealed that gender and age were significant factors in stratifying the results. Among the general population, the anthropometric indicators WtHR and BRI had the highest AUC values of 0.711 (*p* < 0.05) (Table 6). The sensitivity of WtHR in predicting IR was 77.0%, and the specificity was 55.0%. The Youden index of WtHR, which combines sensitivity and specificity, was 0.32. The best cut-off value for diagnosing IR using WtHR was 0.53. Similarly, the sensitivity of BRI in predicting IR was 77.4%, the specificity was 53.8%, and the Youden index was 0.31. The optimal cut-off value for diagnosing IR using BRI was 4.00 (Table 7).

**Table 6:** AUC value of anthropometric indicators in predicting IR.

Variables	All (*n* = 1,592)		Male (*n* = 750)		Female (*n* = 842)		Age < 60 (*n* = 843)		Age ≥ 60 (*n* = 749)	
	AUC	*P-*value	AUC	*P-*value	AUC	*P-*value	AUC	*P-*value	AUC	*P-*value
BMI	0.691	<0.001	0.648	<0.001	0.725^▲^	<0.001	0.730^▲^	<0.001	0.650	<0.001
WHR	0.624	<0.001	0.610	<0.001	0.637	<0.001	0.657	<0.001	0.571	0.002
WtHR	0.711^▲^	<0.001	0.669	<0.001	0.744^▲^	<0.001	0.748^▲^	<0.001	0.655	<0.001
CI	0.641	<0.001	0.598	<0.001	0.671	<0.001	0.675	<0.001	0.581	0.001
ABSI	0.586	<0.001	0.546	0.043	0.614	<0.001	0.603	<0.001	0.538	0.101
BRI	0.711^▲^	<0.001	0.669	<0.001	0.744^▲^	<0.001	0.748^▲^	<0.001	0.655	<0.001
AVI	0.694	<0.001	0.653	<0.001	0.727^▲^	<0.001	0.729^▲^	<0.001	0.644	<0.001

AUC, area under receiver operating characteristics curve. ▲: AUC ≥ 0.700.

**Table 7:** Anthropometric indicators with AUC greater than 0.7.

Indicators	AUC	Sensitivity (%)	Specificity (%)	Youden index	The beat cut off value
General population					
WtHR	0.711	77.0	55.0	0.32	0.530
BRI	0.711	77.4%	53.8	0.31	4.00
Female					
WtHR	0.744	74.9	62.9	0.38	0.540
BRI	0.744	74.9	62.9	0.38	4.20
AVI	0.727	69.1	68.5	0.38	15.14
BMI	0.725	60	77	0.35	24.96
Age < 60 years old					
WtHR	0.748	66.7	71.7	0.38	0.540
BRI	0.748	66.7	71.7	0.38	4.15
AVI	0.729	80.4	55.1	0.36	14.83
BMI	0.730	65.7	68.3	0.34	25.10

WtHR, waist-to-height ratio; BRI, body Roundness Index; AVI, abdominal volume index; BMI, body mass index.

In the stratified analysis of different genders, the AUC values of all anthropometric indicators were generally higher in women than in men. For men, the AUC values ranged from 0.546 to 0.669 (Table 6), while for women, they ranged from 0.614 to 0.744 (Table 6). Among women, the AUC values of WtHR, BRI, AVI, and BMI were above 0.700, indicating a high accuracy in predicting IR. Specifically, the AUC values for these indicators were 0.744, 0.744, 0.727, and 0.725, respectively (Table 6).

When analyzing different age groups, the AUC values of anthropometric indicators among individuals aged < 60 years old ranged from 0.603 to 0.748 (Table 6), while for those aged ≥60 years old, the values ranged from 0.538 to 0.655 (Table 6). In the younger age group, the AUC values of WtHR, BRI, BMI, and AVI were more outstanding than 0.700, specifically, which were 0.748, 0.748, 0.730, and 0.729, respectively (Table 6). The specific values for each anthropometric indicator’s sensitivity, specificity, Youden index, and best cut-off value can be found in Table 7 if the AUC value is above 0.700.

## 4 Discussion

In the 1988 Banting Lecture, Reaven posited that insulin resistance (IR) increases not only the risk of diabetes but also constitutes a core feature of metabolic syndrome (44, 45). The excessive accumulation of body fat is a major cause of IR and is closely related to body shape (46–48). Due to their simplicity and ease of acquisition, anthropometric indicators are increasingly studied in relation to metabolic diseases. However, the correlation between these indicators and IR in the Chinese population still warrants further exploration.

This study included seven anthropometric indicators. WtHR, BMI, WHR are relatively traditional anthropometric indicators already applied in Chinese clinical practice (28, 49, 50). CI, ABSI, BRI, AVI are more recently developed indices with fewer studies and applications in the Chinese population (51–53). Although all seven indicators have been shown to be associated with metabolic syndrome, diabetes, fatty liver, and cardiovascular diseases, their correlations with different diseases vary to different extents, and it seems that each indicator has its own brilliance (28, 54).

Logistic regression analysis in this study demonstrated that the odds ratios (OR) for the seven anthropometric indicators and HOMA-IR were all greater than 1 (*p* < 0.05). This indicates a correlation between IR and these indicators, with the risk of IR increasing as these indicators rise. In predicting IR, WtHR and BRI stood out among the indicators, outperforming BMI, WHR, CI, ABSI, and AVI. The AUC value for WtHR was 0.711, with a critical value of 0.530 and a Youden index of 0.32. The AUC value for BRI was 0.711, with a critical value of 4.00 and a Youden index of 0.31. In females or individuals under 60 years old, the AUC values for BMI, WtHR, BRI, and AVI were all greater than 0.700, indicating good predictive value.

WtHR uses waist circumference and height to assess body fat distribution and is increasingly valued in studies for its importance in predicting metabolic diseases, often showing superiority over other traditional anthropometric indicators such as BMI and WHR (55). In a study on the association between obesity and insulin resistance markers in the United Arab Emirates, WtHR remained positively correlated with insulin resistance even after controlling for BMI (50). In another study predicting hypertension-diabetes risk in the Chinese population, WtHR similarly showed an advantage, outperforming BMI and WC (55). In this study, WtHR outperformed other traditional anthropometric indicators in predicting IR. A possible explanation is that WHtR can better reflect the accumulation of abdominal or ectopic fat in individuals with IR. Compared with general obesity indices, abdominal obesity is a more important risk factor for metabolic diseases (50, 56). Adipose tissue secretes factors that may impair glucose tolerance, cause chronic inflammation in adipose tissue, interfere with insulin signaling pathways, and lead to IR (56, 73). Studies suggest that WHtR, as a phenotypic marker of total fat and regional obesity, can identify individuals with lower body weight but increased ectopic fat accumulation (57). These could be the reasons why WHtR can sensitively identify IR. Related studies have shown that the risk of metabolic diseases is higher when WtHR is greater than 0.500 (34, 58). This study suggests that the threshold for predicting IR is 0.540, the same for females and individuals under 60 years old, which is 0.540. This indicates that thresholds need to be adjusted for different populations.

BRI, created by Thomas (59) et al., is a relatively new anthropometric method that, also considers waist circumference, providing a comprehensive reflection of visceral fat distribution. In this study, BRI’s predictive ability for IR was stronger compared to other indices (except WtHR). Some studies found that BRI outperformed other anthropometric indicators in estimating the risk of various clinical endpoints, including cardiometabolic diseases, kidney diseases, and cancers (60–62). Additionally, longitudinal studies have shown that high BRI is significantly associated with an increased risk of all-cause mortality and cardiovascular disease-specific mortality (63, 64). Considering numerous research results, it is reasonable to speculate that BRI is an excellent anthropometric method for predicting IR and metabolic diseases. In this study, the critical value for predicting IR using BRI was 4.00, with the critical value for females being 4.20 and for individuals under 60 years old being 4.15.

This study also conducted gender and age stratification analyses. In different gender and age stratification analyses, except for ABSI, the other six indicators also showed significant correlations with IR. In terms of diagnosis, BMI, WtHR, BRI, and AVI showed better diagnostic ability in females or individuals aged <60. This study found no significant difference in IR between males and females, but it did observe that specific parameters such as WtHR, BRI, BMI, and AVI were better predictors of IR in females than in males. This difference in predictive accuracy may be due to the higher fat content in females compared to males. Numerous studies have shown that even within the same weight range, females have significantly higher fat content than males (65, 66). This study also found that females had a significantly higher body fat percentage than males through body measurements (as shown in Supplementary Table S1). Additionally, this study observed a significantly higher proportion of IR patients in the ≥60 age group compared to the <60 age group. Age-related chronic inflammation may lead to increased IR, possibly caused by lipid accumulation, adipose tissue or mitochondrial dysfunction, and endoplasmic reticulum stress (67). However, some studies (68) suggest that in males, insulin sensitivity seems to depend more on body fat rather than age, indicating that obesity has a more pronounced impact on IR compared to physiological factors related to age.

In this study, the correlations of the WtHR and BRI with IR were significant, possibly because these two indices better reflect fat distribution, particularly visceral fat. Visceral fat is more metabolically active than subcutaneous fat. Visceral adipocytes secrete inflammatory cytokines and adipokines such as adiponectin and leptin, which can block or interfere with insulin signaling pathways through various mechanisms (69). Additionally, visceral adipocytes tend to secrete more free fatty acids (FFA). FFAs can enter the liver directly, causing hepatic fat accumulation and promoting IR (69, 70). Besides being associated with IR, visceral fat is also a high-risk factor for diabetes, hypertension, and cardiovascular diseases (71, 72).

Both WtHR and BRI are easy to obtain and simple to calculate, making them sensitive tools for identifying IR. They are particularly useful in detecting hidden IR in individuals with lower body weight but increased ectopic fat accumulation. By establishing diagnostic thresholds suitable for the Chinese population, these indices can be applied in health check-ups and clinical practice for predicting IR risk and assessing its severity. In the current routine physical examinations in China, blood tests include venous blood glucose but do not include insulin, making it impossible to calculate HOMA-IR. This provides an opportunity for the application of anthropometric indices. At the same time, the physical examination includes height, weight, waist circumference, and hip circumference, which makes the application of anthropometric indices extremely convenient without adding extra costs or labor. The application of anthropometric indices is also important in the population screening for metabolic disease risk. According to this study, the application of excellent anthropometric indices still needs to consider gender and age factors. Furthermore, calculation apps can be developed to popularize WtHR and BRI among the public, enabling regular self-monitoring and assessment. Based on monitored risks, lifestyle intervention and measures for the prevention and control of metabolic diseases can be guided. This will enhance the general public’s understanding of IR and promote the prevention of metabolic diseases.

## 5 Conclusion

To further elaborate on the conclusion, this study utilized a large sample size to thoroughly analyze the relationship between anthropometric indicators and IR. By doing so, the study successfully identified WtHR and BRI possessed exceptional predictive abilities for IR across the entire study population, particularly among women and individuals under 60. This means that when using WtHR and BRI to predict IR, it is necessary to consider gender and age factors and adopt different diagnostic values.

## 6 Deficiency

In addition to notable findings, it is also crucial to acknowledge the limitations of this study. Firstly, other potential confounding factors, such as dietary habits and levels of physical activity, disease history, family history, were not considered. This study used a non-random consecutive sampling method for enrollment, and compared to the random stratified sampling method, the possibility of bias cannot be ruled out. Finally, the impact of COVID-19 on case collection was not taken into account. Based on the preliminary findings and identified limitations, it is hoped that future research can conduct cohort studies with larger sample sizes in the Chinese population to validate and further apply these research results.

## Data Availability

The original contributions presented in the study are included in the article/Supplementary material, further inquiries can be directed to the corresponding authors.
