# Supplementary material for: Waist-to-height ratio and body roundness index: superior predictors of insulin resistance in Chinese adults and take gender and age into consideration
Source: Front Nutr. 2024 Dec 18;11:1480707. doi: 10.3389/fnut.2024.1480707 (PMC11688232; doi:10.3389/fnut.2024.1480707)
Supplement: Supplementary file 1 [file Data_Sheet_1.docx]

**Schedule 1 Comparison of main indicators in different gender and age**

|  | Gender | | |  | Age | | |
| --- | --- | --- | --- | --- | --- | --- | --- |
|  | Male | Female | P-value |  | Age＜60 | Age≥60 | P-value |
| Age | 55.44±14.20 | 57.70±14.92 | ＜0.05 |  | - | - |  |
| Gender:F(%) | - | - |  |  | 399(47.3%) | 438(59.0%) | ＜0.05 |
| BFP | 26.38±4.97 | 32.38±6.20 | ＜0.05 |  | 28.39±6.33 | 30.86±6.22 | ＜0.05 |
| VFI | 12.29±4.78 | 8.97±4.48 | ＜0.05 |  | 10.22±5.27 | 10.83±4.42 | ＜0.05 |
| BMI | 26.00±3.66 | 25.13±3.88 | ＜0.05 |  | 25.87±4.17 | 25.15±3.30 | ＜0.05 |
| WHR | 0.965±0.073 | 0.927±0.090 | ＜0.05 |  | 0.935±0.086 | 0.956±0.082 | ＜0.05 |
| WtHR | 0.554±0.056 | 0.560±0.069 | 0.091 |  | 0.546±0.066 | 0.570±0.057 | ＜0.05 |
| CI | 1.30±0.08 | 1.29±0.10 | ＜0.05 |  | 1.27±0.09 | 1.33±0.09 | ＜0.05 |
| ABSI | 0.083±0.080 | 0.082±0.104 | 0.101 |  | 0.081±0.005 | 0.085±0.006 | ＜0.05 |
| BRI | 4.50±1.19 | 4.64±1.46 | ＜0.05 |  | 4.34±1.37 | 4.84±1.26 | ＜0.05 |
| AVI | 18.11±3.77 | 15.98±3.72 | ＜0.05 |  | 16.81±4.29 | 17.18±3.37 | 0.055 |
| HOMA-IR | 3.46±3.28 | 3.35±3.22 | 0.508 |  | 3.30±3.25 | 3.53±3.25 | 0.159 |
| IR (%) | 511(68.1%) | 550(65.3%) | 0.235 |  | 535(63.5%) | 524(70.6%) | ＜0.05 |

Abbreviation: F: female, BFP: body fat percentage, VFI: visceral fat index, BMI: body mass index, WHR: waist-to-hip ratio, WtHR: waist-to-height ratio, CI: conicity index, ABSI: a body shape index, BRI: body roundness index, AVI: abdominal volume index, IR: insulin resistance.


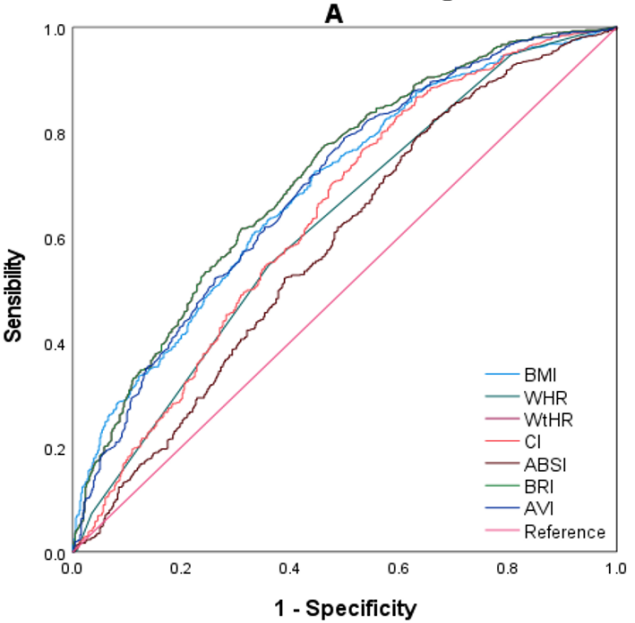

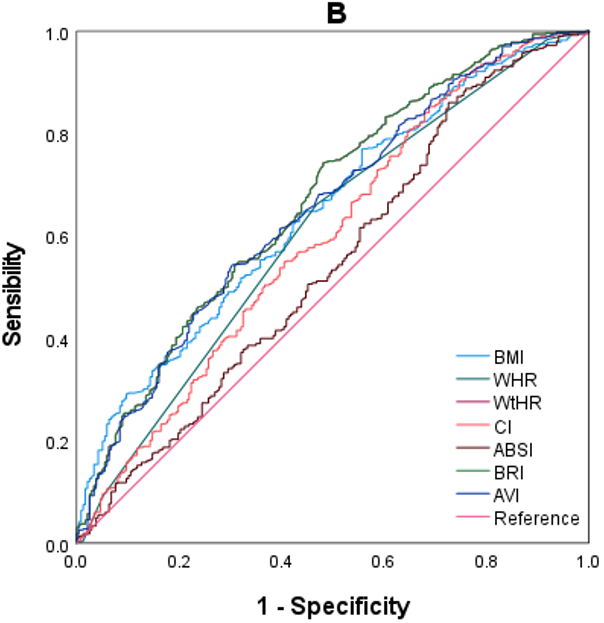

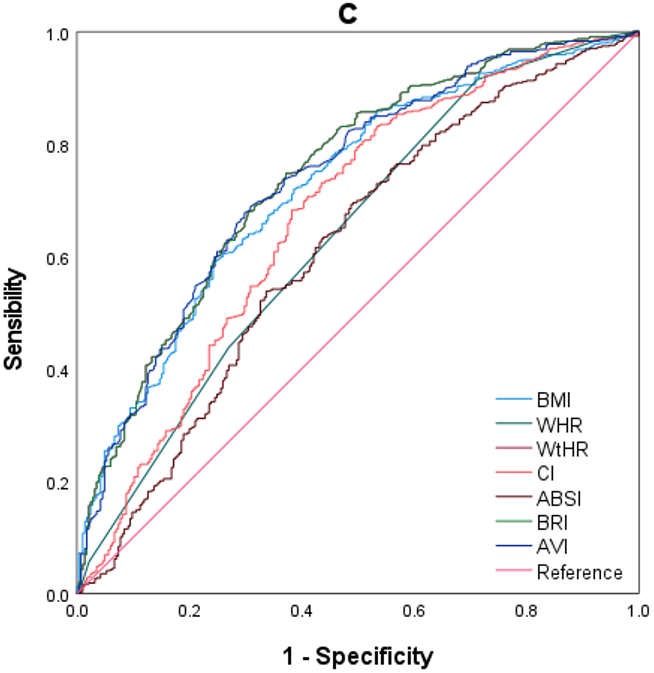

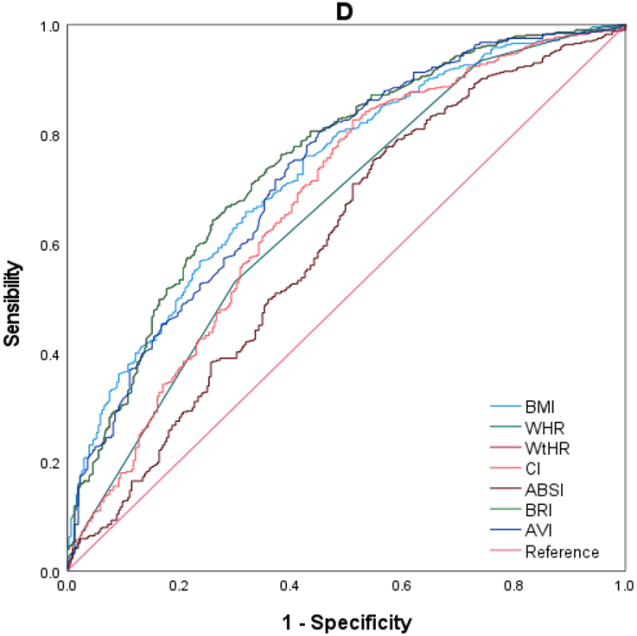

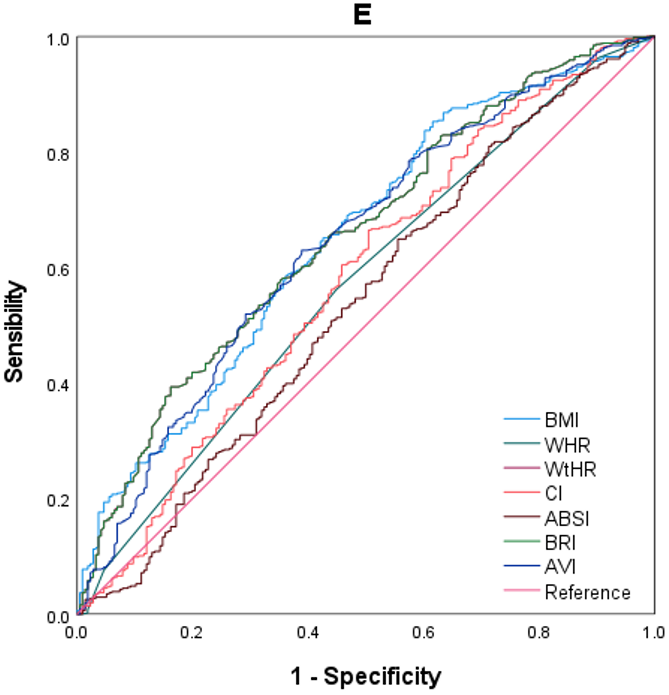


Supplementary figure 1 ROC curve of anthropometric indicators to predict IR

(A: ROC curve of anthropometric indicators to predict IR in the overall study population. B: ROC curve of anthropometric indicators to predict IR in males. C: ROC curve of anthropometric indicators to predict IR in females. D: ROC curve of anthropometric indicators to predict IR in age ＜60. E: ROC curve of anthropometric indicators to predict IR in age ≥ 60. Abbreviation: ROC curve: receiver operating characteristic curve)
